# Fostering learning capacities for meaningful, healthy and efficient studying in undergraduate medical education: evaluation of a longitudinal learning workshop

**DOI:** 10.1186/s40359-021-00631-5

**Published:** 2021-09-02

**Authors:** Miriam Thye, Diethard Tauschel

**Affiliations:** 1 Faculty of Health, Department of Medicine, Integrated Curriculum for Anthroposophic Medicine (ICURAM), Witten/Herdecke University, Witten, Germany; 2 Faculty of Health, Department of Psychology and Psychotherapy, Witten/Herdecke University, Witten, Germany

**Keywords:** Learning, Learning life, Undergraduate medical education, Medical students, Didactics, Qualitative research, Self-regulated learning, Self-determination, Self-care

## Abstract

**Background:**

Academic studies place high demands on the development of learning capacities. Beyond learning techniques, knowledge about the effect of the learning environment, as well as the ability for self-regulation, self-determination and self-care play a major role in the development of learning skills. A longitudinal learning workshop was developed aiming to support academic learning life. The study at hand describes and evaluates this intervention.

**Methods:**

Students participated in a seven-week program fostering reflection and training on physical, physiological, psychological and mental dimensions of learning. Fifty evaluations of medical students reflecting the workshop underwent qualitative analysis of open-ended questions concerning changes students experienced in their learning life. In addition, general satisfaction was measured quantitatively.

**Results:**

Qualitative results revealed an impact on five core dimensions of students´ learning life: knowledge gained about the process of learning, enhanced awareness of intrapersonal learning processes, getting easier into action, experience of change and raised skills of regulating one´s learning behavior. Students evaluate the workshop as helpful, supportive and as a source of guidance. Quantitative results demonstrated good overall satisfaction with the intervention.

**Conclusions:**

Educating knowledge about learning how to learn and providing skill training of how to regulate physiology, psychology and mentality should be taken into account in order to support the multidimensional learning life of students. Using a holistic, anthropologically grounded approach could be considered to enhance healthy, meaningful and efficient ways of learning. This learning workshop seems to be a useful and transferable tool to support students’ development of learning capacities.

**Supplementary Information:**

The online version contains supplementary material available at 10.1186/s40359-021-00631-5.

## Background

Medical studies and the formation of a physician identity place high demands on the development of comprehensive and efficient learning skills. Learning is not only about acquisition of knowledge and preparation for the next examination; learning is a lifelong reflective and adaptive process of change and formation [1]. A fundamental element of learning is the learning environment [2]. This involves specific surrounding factors such as a quiet work atmosphere, social support, as well as self-regulation in terms of daily learning periods. Such a learning approach—which facilitates the perception of knowledge acquisition not as superficial, but as a thorough and long-term process leading up to transfer—also includes factors of health and life-style, like sufficient sleep, exercise and diet [3]. Therefore, health plays an essential role in connection with a student’s learning environment.

Medical students and doctors report deficits with regard to health, meaningfulness, and joy [4], as well as poor quality of life [5]. Medical students’ perceived stress and emotional distress levels are high compared to the general population [6]. Described possible consequences are loss of empathy, cynicism, burnout, depression, somatic illnesses, or substance abuse [7–9]. Every second medical student experiences a burnout during the course of medical training [10]. In a systematic review, Mata et al. describe a prevalence of depression between 20.9% and 43.2% in young physicians which is high compared to the peer group. The authors report instances of existential crises and numerous problems during medical studies [11]. Molodynski states that an increased prevalence of poor health varies in accordance with the cultural context, but nevertheless constitutes an international phenomenon among medical students [12]. Cognitive performance and learning capacities depend heavily on physical and mental health [13], and impaired mental health in particular affects academic learning performance [14].

Higher education institutions thus have the task of enabling students to meet the demands of their studies and careers with a broad-based qualification for learning and health promotion. This leads to the development of a profound, demanding, and sustainable learning personality (“sophisticated learners”) [15]. Abilities to assume responsibility for, and regulate, one’s own studies should be built up in the course of medical training [16]. Intrinsic motivation and curiosity are further determinants of successful learning and should be encouraged [17]. Young doctors in particular describe the experience of meaningfulness in what they do as a key element of burnout prevention [4].

As described by Ayalya, self-care includes nutrition, physical activity, interpersonal relations, spiritual growth, health responsibility, and stress management. Medical students with higher resilience report improved quality of life and a better perception of their learning environment [18]. The development and promotion of resilience is therefore a valuable strategy to reduce emotional stress and improve medical training [19]. Thus, mental health programs, mind-body skills education, and training programs [20] are increasingly introduced in medical curricula [21–24]. Although showing some effects, the focus of these interventions has been exclusively isolated to one or a few aspects of learning that need to be strengthened. Apart from lack of self-care, students report problems with setting specific learning objectives due to aimlessness, flawed time management, insufficient learning strategies, little self-evaluation and absence of self-motivation; in addition, a lack of intrinsic interest in course content is an obstacle to successful learning [25]. All of these are key competences for self-regulated learning [26], which should be taught for long-term learning and comprehension as they constitute the determinants of successful studies.

This study evaluates a seven-week longitudinal learning workshop (LW). It has been designed as a holistic model to become aware of and handle factors that influence learning and empower the entire, multidimensional learning life. The broad idea is to educate students how to approach learning holistically and to facilitate learning with increased self-regulation. An adapted and expanded version of the model of self-regulated learning (SRL) as a tool of long-term knowledge acquisition [27] is used as the basic concept for processes and didactics. The model is available on request from the first author (MT). The approach of the LW is to combine specific learning techniques with education on how to improve the learning environment [2, 3], consider health, meaning, and efficiency, and (re)discover the joy of learning. The LW conveys specific expert knowledge on learning; learning techniques and strategies are presented, individually analyzed and practically trained; individual learning environment and intrinsic motivation for learning are considered. Participants explore the meaning they personally attach to their studies, since learning as a thorough, sustained and lasting process requires that the matter to be learned is of significance to the learner [1]. The didactic concept of the LW therefore relies on diversity of didactical approaches. It comprises keynote speeches, reflection, individual and group work, as well as homework. The focus is on defined tasks, including the transfer of skills into daily activities, the reflection of the achieved personal efficiency and the possibility of a permanent adaption of new skills and tools into students’ learning life. The workshop’s concept is founded on a pragmatistic learning theory [1] and theories related to learning: principles of the organism, psyche, and the self (adaptive normalization [28]; effort-recovery theory [29], self-determination theory: [30], as well as holistic (medical) anthropological models [31–33]). The training seeks to offer insights into physiological, psychological and mental fields known to contribute to an improved learning environment and to the development of adaptive learning techniques. It does so by taking a holistic approach and by addressing several relevant competencies for adaptive learning—self-reflection, self-regulation, self-care, self-management, and self-development. For a full overview of the topics addressed during LW see Table 1.

**Table 1:** Overview of the seven-week longitudinal learning workshop

Topic	Competency	Content	Examples
physical	Gaining knowledge on the physical determinations of learning	Nervous system, neuronal plasticity, access ways to content and memorization, learning theories, learning environment, health and learning	Usage of diverse access ways, spaced repetition, Mnemo techniques, ratio time spent self-studying /instructional time, work place and learning conditions
physiological	Benefit and regulation of the resources of the organism	Self-care and health, chronotype, sleep, taking breaks, exercise, nutrition, abuse of stimulants	(self-)study time in accordance to own chronotype, sleep length and sleeping times, power nap, weekly rota, study-life-balance with recreational time, body exercises
psychological	Benefit and regulation of the psychological resources to improve learning	Intrinsic & extrinsic motivation, procrastination, fear, excessive demands, collaborative learning, concentration, mental hygiene, time management	Learning with partners, social support, digital detoxing, getting things done, regulation of thoughts—emotions—volition, treasure chest
mental	Self-realization to gain insight, profit and regulation of mental resources	Self-reflection, appeal, over-all goal/mission, spirituality, awareness and realization of attitudes, sense-making, managing meaning	Reflection and adaptation, learning diary, learning coach, time for own questions and goals, inner appeal, meditation & mindfulness, SMART- goals and objectives

To the best of our knowledge, no didactic intervention has been reported so far that can be compared to the holistic approach of the LW. No other intervention known to us tries to explore and foster such a broad spectrum of learning life. Accordingly, no qualitative analysis describes the experience of medical students of such an intervention. The aim of our study is to reveal the immediate effects of the LW on students’ learning life. An explorative-evaluative approach was chosen for this purpose without pre-formulated hypothesis. The focus of this study is the identification of regularities based on reflection and evaluation [34], and thus on students’ life realities.

## Methods

### Setting

In 2011, the LW was introduced as an elective longitudinal course with in the context of *Studium Fundamentale-* a competence-oriented range of additional studies in humanities, arts, philosophy and other fields that are obligatory for students of all disciplines at Witten/Herdecke University [35, 36]. The development of the LW was a joint venture of staff and students. Students participated in the conceptual design, as well as in implementation, organization, and teaching [37]. The longitudinal LW structure comprises one intensive day in class (8 h) and six subsequent meetings of 90 minutes each on a weekly basis; the schedule may vary in accordance with semester planning. The LW concludes without a specific examination, but proof of performance can be issued to gain ECTS *(European Credit Transfer and Accumulation System)*. Attendance in >80% of the course was mandatory to gain credit points. More information about the concept and the content of the LW is available upon request from the first author (MT).

### Study design

An explorative qualitative research design was chosen to reach out for the nature of a phenomenon without having formulated any hypothesis. The aim is to get direct insight into the experiences of the participating students and to understand how the phenomena manifests in different perceptions [38].

The authors followed the *Standards for reporting qualitative research* (SROR) in presenting and structuring the research question, data collection, sampling, data analysis and conclusions resulting therefrom [39]. Earlier theories and models on learning are duly considered, but do not dominate, the research process, analysis, or conceptual design. Procedures are inductive and take the form of an iterative research process including regular validation, expansion, and cross-checking by various other researchers.

For the survey, the authors used the Qualitative Short Survey (QSS) method. This instrument of intra-method mixing permits a meaningful combination of qualitative and quantitative data (triangulation). In this manner, subjective findings can be evaluated with the help of quantitative survey data, participants can be assessed individually, groups can be compared, and elaborate statistics can be produced [40].

This type of survey has the following advantages for the present study and the available human and financial resources:

- meaningful format of evaluation which ensures anonymity and reduces distortion in response behavior caused by social desirability, compared to personal in-depth interviews [41]
- allows comparison between different subgroups [42]
- permits time efficient on-site evaluation immediately following the seminar, with a higher response rate than online evaluation at a later time.

### Questionnaire

Assessments by LW participants underwent analysis, and the questionnaire used in this case was specifically designed for evaluation of the LW. It comprises 4 pages with sociodemographic questions, 13 semi-quantitative and three open-ended questions, as well as a self-assessment of one’s own abilities and an overall rating.

For the purposes of this study, three questionnaire elements (Additional file 1) are analyzed asking for a reflection on the development of the respondent’s learning behavior and reveal direct information about medical students’ learning life.

We used two open-ended questions with the option of free-text answers:

1. *Have you noticed any changes in your learning life due to your participation in the LW? If yes, to what extent?*
2. *How do you evaluate this change in general? (Satisfaction)*

And thirdly, a rating on a quantitative scale:

1. *The overall grade I give to the LW is (1–6): ___*

*(German system of school grades: (1)* = *Very Good; (2)* = *Good; (3)* = *Satisfactory; (4)* = *Sufficient; (5)* = *Poor; (6)* = *Deficient)*

#### Data collection

Questionnaires were distributed to all participants at the end of the last session of the LW, immediately filled in by students and collected subsequently. Students were informed that participation in the evaluation was voluntary. Completion of the entire questionnaire took between 12 and 18 min approximately.

### Ethics approval and consent to participate

Data collection was anonymous. Students were advised that completion and return of questionnaires were voluntary, and that non-participation in the evaluation had no negative consequences for attendance at the LW nor potential proof of performance. Participants were free to decide how much time to invest in completing the questionnaire, how profound, detailed and precise their answers should be, and how much personal information they were willing to give. Thus, informed consent was obtained from all subjects involved in the study. A vote of ethical approval was obtained from the Ethics Committee of Witten/Herdecke University (submission no. 205/December 2019). So, all methods were carried out in accordance with relevant guidelines and regulations.

### Sample

Available evaluations from 2011 up to 2019 form the basis of this study. In this period of time, the LW was conducted 17 times. Due to our focus on medical education, only evaluations made by medical students were included in the study. Those by students of other disciplines as psychology, dental medicine, economics and cultural studies were excluded for the study at hand.

Fifty students filled in the evaluation of whom six did not enter their demographic data, but did complete their evaluation. Their answers were included in the qualitative analysis. The response rate was 30.67%. The average age was 22.5 years (between 19 and 30 years of age). Participants (28 f/16 m) had completed 2.7 semesters on average (between 1 semester min. and 9 semesters max.). Responses were heterogenous in terms of length, form, and depth. Some evaluations were short lists of key aspects. Other participants wrote long paragraphs using full sentences. All transcripts were translated and back translated from German to English for publication purpose.

### Data analysis

Three student assistants and the author (MT) transcribed the answers given in the questionnaire. The basic technique used to analyze the data is the qualitatively oriented content-analytical method of structuring content analysis with subsequent frequency analysis in adherence to Mayring [43]. Following this method, an iterative step-by-step procedure served to inductively develop a system of categories. The authors used the computer software *MAXQDA2018* to analyze complex non-numerical data [34]. The software *SPSS Statistics 25* was used for the quantitative analyses for research question number three and the descriptive demographics statistics.

The content-analytical criterion of inter-coder reliability was applied to test the reliability of qualitative findings. Data was comprehensively analyzed by a researcher who is experienced with the method (MT) and two students (MB & RS) who assisted in the design and the conduct of the LW. They analyzed a randomized excerpt of the data to permit the inclusion of the student perspective on the findings in the process of analyzing and interpreting results. Two other researchers, familiar with the method, encoded up to 50% each of the entire data material (DT & FA). Assignment of material was randomized. As a consequence, the coding-team was a heterogenous group of various researchers with different personal and professional experiences and preconceptions resulting therefrom [44].

The reliability of the dataset derives from the time of the measurement (17 realizations within a period of eight years), the sample size (N = 50) and the setting of the workshop (longitudinal, attendance check to gain ECTS, students’ involvement).

Firstly, the reliability of the analysis derives from the transparency of the process (by depicting every step in detail, the analysis becomes comprehensible and assured, that the results are valid and reliable). Secondly reliability of qualitative research is high, if intersubjectivity (plausible description of the iterative rating process and presentation of the results with reflection) is achieved [38, 45]. The five encoding schemes were disclosed, presented, jointly considered, and discussed in an inter-coder-reliability-team meeting. The researchers specifically tried to identify divergencies in the interpretation of terminology and thus to obtain a clear picture of the respective degree of subjectivity in such interpretations [46]. Excerpts from the material were studied together to see whether essential qualities of students’ statements had been appropriately and congruently summed up in encoding. The emerged encoding schemes coincided; incongruous notions were defined more distinctly. Discussion topics were conclusiveness, precision, and logical congruence in the definition of the encoding schemes and encoding guidelines. Congruent and contradictory elements of the five coding schemes were debated, and mutually agreed definitions were established. Based on this the definitions a final encoding scheme and encoding guideline were finalized by MT and used in the further elaboration of the model and the analysis [43].

## Results

### Qualitative survey

The structuring qualitative content analysis [43] yielded interesting results. Students experience an impact on five core dimensions of their learning life: **knowledge** gained about the process of learning, enhanced **awareness** of intrapersonal learning processes, getting easier into **action**, **experience** of change and raised skills of **regulating** one´s learning behavior. Notably, none of these dimensions was mentioned distinctly more often or predominantly, and therefore is of greater relevance compared to the others. Table 2 illustrates the code groups with pertinent definitions, corresponding encodings, and the identified frequencies:

**Table 2:** Overview: Perceived dimension of impact and core aspect of the results

Dimension of impact	Core aspect	Percentage distribution (%)
Dimension of knowledge	I have acquired new knowledge about learning	23
Dimension of awareness	I have become aware of something	18
Dimension of action	I try out other ways of learning for myself, under supervision and/or together with others	18
Dimension of experience	I experience change in my learning life	24
Dimension of regulation	I act with intention to achieve the goal I have set myself	17

### Dimension of knowledge

*I have acquired new knowledge about learning.* Participants reported that attendance resulted in additional theoretical knowledge about learning which they perceived as helpful, guiding, and supportive. A total of 23% of all references concern this dimension. More than half of participants (62%) mentioned aspects which are associated with the knowledge dimension. Students reported that this additional knowledge made their learning more efficient and effective. Key terms mentioned in this context were *various structuring learning techniques, power napping, weekly schedule, mind mapping, treasure box (award), meditation, rhythms,* and *the significance of joy in learning,* as well as *intrinsic motivation*.

*“The LW has brought about major changes in the way I learn.*

*I have received helpful suggestions for learning methods which I have put into practice. (…).*” *(*Participant no. 36)

### Dimension of awareness

*I have become aware of something.* Students described positive developments on this dimension due to the LW. The reasons provided were growth in theoretical knowledge and the assignments to be addressed on the one hand, and on the other, the exploration of one’s own attitude, motivation and learning environment. What emerged from these reflections was an enhanced awareness of the personal learning processes. The core elements named by participants were: *reflection of learning preferences, questions of meaning, the self, one’s own learning behavior, contemplation, self-care*. Specifically, joint reflection with others is described as meaningful. This dimension accounted for 18% of references, and 66% of participants rated experienced changes at this dimension as relevant.

*“The LW helps me to remain true to myself in learning. This gives me strength and structure and also a lot of pleasure in shaping my ways of learning and my daily life in general.*

*intentional learning means that I organize my learning proactively and do not just get started. This way is more fun, I feel structured and more myself and more efficient, and therefore more motivated*

*- I pay attention to sleep and get up in the morning feeling fresh. What’s new is that I get up earlier but nevertheless well rested. Every day around noon I take a nap for 15 minutes, which I look forward to and which gives me lots of energy.*

*- I have an excellent weekly schedule which I admit I haven’t followed as strictly as earlier in the semester. Power and motivation for organization and activity tend to decline. Now I am looking forward to the Christmas break.*

*- Obviously, it takes effort and energy to become proactively engaged. I did not do much of that before.”* (Participant no. 127)

*“With a focus on my motivation, learning has become easier and more of a pleasure!” (*Participant no. 74)

### Dimension of action

*I try out other ways of learning for myself, under supervision and/or together with others.* Students tested various learning methods collaboratively during the seminar under supervision, and jointly discussed individual experiences with their peers. Participants report that in this manner the LW opened up new options for action. Positive key terms in this context were: *effectiveness, productivity, concentration, learning group* and *regular breaks. Obstacles* to be faced at this dimension are *implementation problems* and *lack of discipline*. A total of 18% of the responses refer to the dimension of action. More than half of the students (64%) report changes at this dimension triggered by the LW. Exchange with fellow students was specifically mentioned as a helpful element.

*“ I tried new ways of learning. 1. I discovered mindmapping for example as a good tool for me, which I knew before LW but didn’t really like. 2. We created a learning team and tried to learn collaboratively. 3. I minimized my physical personal attendance at lectures. 4. I tried intercommunication with students from higher semesters. The LW supported that. 5. I started reflecting on my personal learning behavior: What is good and what is bad? 6. I learned new ways of pre- and postprocessing of the lectures and the problem-based-learning-cases. That helped me a lot with remembering and aided recall.”* (Participant no.4)

*“Exchange with other course participants has given me a feeling of safety. It was reassuring to hear from others in the plenary session that they basically grapple with problems/difficulties in learning similar to my own. That was really good to know.” (*Participant no. 16)

### Dimension of experience

*I experience change*. Participants described several areas of practical experience as triggers of change. Most quoted is the dimension of emotions/feelings for negative as well as positive experiences, or changes in affect. Students report enhancement received in the LW to reconsider their learning approach. The individual nature of responses referring to this dimension was interesting to note. Key terms in this context were: *enjoyment, courage, motivation, anxiety, self-confidence, insecurity, health, sense of well-being, more positive attitude* and *stress reduction*. Most students (68%) report that they registered changes in experience, feelings and attitude as a consequence of the LW. A total of 24% of all responses referred to this category.

*“My attitude towards learning and motivation to study have improved as well. Now learning is more fun, and I repeatedly remind myself that studying is what I want to do and what I enjoy, and not just misery. My test anxiety is not as bad as before. I will definitely follow the advice how to manage the test situation better (e.g. by bringing objects which improve well-being). I am really glad and grateful for this seminar; it was immensely helpful to optimize learning time and learning performance.”* (Participant no. 36)

### Dimension of regulation

*I act with intention to achieve the goal I have set myself.* Participants described the newly addressed capacity for self-regulation as an important trigger for change. Interesting to mention was, that students characterized self-regulation skills as the primary key to perceived changes at all other dimensions. The dimension of regulation could be called a superordinate dimension in relation to dimensions 1 to 4. It could therefore be assumed to have a broader basis than indicated by 17% of all responses and 60% of respondents, since the capacity for regulation is implicitly contained in the aspects described for dimensions 1 to 4. Key terms were: *regulation of learning periods, time management, time out* and *sleep (adaptation*). These are the most quoted aspects (36 responses). *Digital detoxing* (i.e. more deliberate and regulative use of digital media) is a further aspect mentioned in more recent surveys.

*“I have put some suggestions into practice, and I do no longer feel controlled by stress but rather in control of what I am doing.”* (Participant no. 24)

*“Now I have got more structure, I know how to tackle a problem and not to panic. (…) I would like to establish these learning techniques more firmly and make a habit of sticking to the weekly schedule.”* (Participant no. 74)

The importance of the dimension of regulation is shown again in the fact, that this dimension was the only one where students registered negative experiences as well. But it was not the subject matter of the LW which was perceived as negative; rather, students report a negative affect which they specifically perceived at the second (awareness), third (action), and fourth (experience) dimensions. Students indicated that the LW has helped them to develop an awareness of their own limitations in regulatory skills; it is frustrating for them to see that they are unable to use these skills to the desired extent. Other private reasons were also given for negative experiences, such as *prolonged illness* or *mobbing by fellow students*.

*„(…)a more deliberate approach in my learning behavior/assessment of my learning strategies*

*- have been given a lot of tools and have to try them out *→ *do no longer feel so overwhelmed because now I know lots of options how to tackle the matter. What is still lacking is the courage to actually apply these strategies (…) I feel that I am able and willing to change some things. Some things have already changed, and I like that, but I have not yet reached the point where I can say I have got a satisfactory learning strategy.” (*Participant no. 26)

## Summary of results

In total, the LW had a fundamental and comprehensive impact on various levels of learning. Students evaluate the workshop as helpful, supportive and as a source of guidance. Participants could precisely describe effects and also triggers for the changes they register in their own learning life. No respondent left questions unanswered. Nobody reported any declining or negative trends in learning as a consequence of attending the LW.

Individual students reported frustrating realizations about deficits in their own learning behavior which the contents and exercises of the LW helped them to address:

*“I have started to learn. Looking back to the past semesters, I somehow muddled through, and I realized that this is not really an option (result: failed MEQ1 – MEQ2). What I took home from the LW were many “small” things, techniques and suggestions, e.g. a fixed learning schedule, power napping, continuous repetition; I also realized that other techniques do not really appeal to me (such as mind mapping). I would say that my way of learning has become more regular, comprehensive, consistent and effective, and I feel better with that. But there are still some aspects that I need to optimize.“* (participant no. 10)

Note from the author: At Witten/Herdecke University, the 1st medical state examination is replaced by three MEQ tests (*Modified Essay Question Test*) in combination with two OSCE exams (*Objective Structured Clinical Examination*), pursuant to the pertinent clause in licensing regulations (ÄAppO) on medical model curricula.

### Satisfaction

Students report many changes they have experienced in their learning life. In response to the question whether participants are satisfied with the changes they perceive, 84% (42 out of 50) say they are satisfied. 6% (N = 3) do not respond. 2% (N = 1) say *Yes and No*, and 10% (N = 5) say they are not satisfied. But the reasons given by 80% of these unsatisfied participants (N = 5) reveal that their lack of satisfaction refers not to content or format but to their own regulatory deficits. It appears that they perceive seminar contents as meaningful but they have not found the strength to put them into practice. One dissatisfied participant did not give any further information.

#### Quantitative results

Quantitative results demonstrated good overall satisfaction with this longitudinal intervention. The overall evaluation is based on the German system of school marks (1 = very good/top mark, 6 = deficient/bottom mark) and aims to interpret medical students’ assessment of changes resulting from their participation in the LW.

**Fig. 1:**
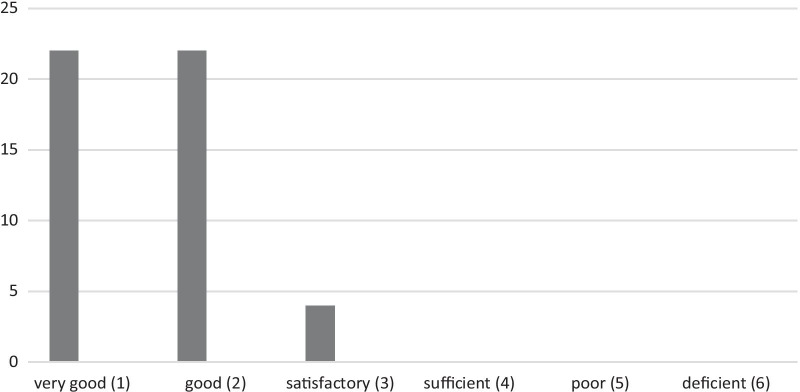
Overall Satisfaction with the Learning Workshop

Fifty students filled out the questionnaire. Two respondents (N = 2) did not provide an overall mark. The LW is generally rated as “good” (median = 2.00; mean = 1.63). The statistical spread of results ranges from “1=very good” to “3=satisfactory” (IQR 1.0; SD 0.64). As Fig. 1 depicts, the distribution of marks is skewed towards good marks—most students rate the LW as very good and good. Only a few of the ratings give 3 as an overall evaluation. A mark below “satisfactory” was not given.

## Discussion

Students who attend the LW report experienced changes on five core dimensions: knowledge, awareness, action, experience and regulation. It is interesting to note that the dimension of regulation or rather the *empowerment for self-regulation* seems not to be on the same level as the other four dimensions, as regulating is a higher capacity. Findings reported by Schunk and Zimmerman [26] indicate that this capacity for regulation, combined with—for example—precise targets, self-evaluation, intrinsic interest, and adaptation of learning strategies, is a fundamental pre-requisite for meaningful long-term learning and thus for successful academic studies. In addition of being a pre-requisite as described, we question if the first four above mentioned dimensions are necessary pre-requisites that contribute to the growing of this regulating capacity, and therefore it would be fundamental that they are addressed in order to promote the development of the (self-regulated) learning capacity of students.

*Self-determination* is another key issue for students. The three basic psychological needs—competence, social integration, and autonomy—inherent in the Self-Determination Theory (SDT [30]) are reflected in the answers given by participants in our study. According to Deci and Ryan, the fulfilment of these needs has an immediate motivating impact on creativity, problem-solving abilities, staying power and the accompanying sense of well-being [30]. If these needs are not met, students may feel frustrated and less motivated, and ultimately experience a decline in well-being and health, which can in turn results in an absolute lack of drive. As to improvements in the learning environment, they mention—among other things—scope for personal initiative, comprehensible reasons for the subject matter to be addressed, waiver of performance comparison or performance-related rewards, and avoidance of overstrain and underchallenge. Participants’ responses indicate that the LW stimulates their basic psychological needs outlined in the SDT, with positive effects on process-orientation as well as associated motivational aspects of learning. It conveys knowledge about learning per se, encourages active participation, trying out, reflecting and adapting, and therefore the development of learning skills (competence). It brings students together inter- and intra-professionally and at different levels of their studies, and invites participants to collaborative exploration and reflection (social integration). In addition, the LW permits students to decide for themselves which learning suggestions to implement in the practice of their individual learning behavior (autonomy).

The concept of *Learning environment* refers to the entirety of study conditions. These comprise amongst others students’ social networks (participation in social life), and their intra-psychological world with all emotions as fear, beliefs etc. involved (*environment* [3, 47]). The answers provided by participating students in this study underline that it is necessary to integrate these learning environmental aspects into the metacognitive process of learning how to learn. Students’ health as a basic element of learning capacity should be strengthened, and resilience should be promoted to ensure long-term successful learning [14, 19, 48]. We know for example. that intrinsic motivation and curiosity [17], a sense of meaningfulness [4] and self-care [18] are important determinants of successful learning. Beyond that, the individual *learning life* could be taken into account if students are to experience the life period of their studies as efficient, healthy and meaningful in the long-run. Learning life can be defined as the sum of all the factors in daily life influencing every student’s daily academic performance. The individual physiology, body, psychology, mentality and spirituality should be worthy to taken into account on the dimensions of knowledge, awareness, action, experience and regulation when aiming to augment learning. It appears to be important in the didactics of medical education to highlight and address all dimensions of the learning life, the environment, and the close relations between studies and private life [49]. Moreover, individual learning behavior is characterized by interaction between various areas of life as the personal, social, biological & the psychological area [25]. The participants of the study at hand depicted the significance of this interaction in detail. Results show that the issues addressed in the LW stimulate significant processes of change in students.

### Strengths and limitations of the study

Strengths and limitations of the research process and the findings are as follows: Qualitative Short Survey (QSS) was used to collect the data for the study. This instrument of intra-method mixing permits a meaningful combination of qualitative and quantitative data (triangulation [40]. The QSS is an expedient format of evaluation which ensures anonymity and reduces potential distortion in response behavior caused by social desirability, compared to personal in-depth interviews [41]. It is, however, less detailed and differentiated than interviews. However, the method served to generate a large number of participants and provided a wide view of students’ personal experiences. Further research could use qualitative in-depth interviews or focus-group interviews as suitable instruments to further deepen and differentiate the insights gained in this manner.

Above that, numerous steps were taken to improve the reliability of results gained by the qualitative methods. However, it is important to note that the process of interrater validation revealed differences in the use of key terms such as *effectiveness* and *efficiency*. Application of these terms was congruent in some questionnaires, and divergent in others. Some respondents left the definition open; others used the terms incongruently within their reply. For further considerations of the coding model development, the authors agreed on the following definitions, in adherence to definitions by Dyckhoff and Souren [50] and adapted to learning contexts of tertiary education: effectiveness stands for “I can achieve my goal and know the required ways and means”, whereas efficiency corresponds to the basic assumption “I can achieve my goal with as few resources as possible”. These definitions have been agreed upon and maintained by all researchers involved for subsequent activities. The scheme is not 100% precise even after good validation, but suitably reflects the realities of student life.

In the overall interpretation of the results, it should be noted that the time frame chosen for the survey right at the end of the LW could be too short to make accurate statements about profound and lasting changes [51]. This limitation, however, is also a strength since memories are still fresh at that time, and therefore relatively unaffected by cognitive distortion. Further research could focus on the long-term experienced changes to find out, in how far they endure over time and foster academic learning in the long run e.g. by using a cross-over-design. A qualitative survey on long-term changes using interviews could be conducted e.g. six months after the end of the longitudinal seminar and might serve to obtain further insights. Moreover, the results at hand depict students’ experiences of a LW at one single university. Interesting research could be to compare the results at hand to experiences made by students following different didactical courses at various places by a broad literature review and comparative studies. A prospective experimental interventional study could be designed to evaluate and compare further impact of didactical approaches on the learning life. In order to understand cultural differences in how to study meaningful, healthy and efficient could be explored by a cultural comparison study.

## Conclusions

This study investigated an elective longitudinal learning workshop with an anthropologically grounded **multidimensional approach to foster learning** capacities. Semi-quantitative results demonstrated good satisfaction with the workshop; qualitative results revealed the impact on five dimensions on medical students´ progress of learning capacities: **knowledge, awareness, action, experience and regulation**. Findings from this study were directly generated from medical students’ perceptions and experiences. Resulting implications provide interesting suggestions for the curricular design and development of medical education. It appears that a holistic, methodological, and thematic approach of this type has not yet been sufficiently developed or rather included in the exploration of the contemporary learning culture. Influencing academic performance by addressing the entire ***learning life*** processes of change at physiological, psychological, and mental levels) should therefore be considered as a concept of some relevance for further efforts in supporting students’ academic learning in future research. This study is intended as a first step in a new direction of understanding the concept of learning and a basis for further research activities and publications.

The LW may serve as a model example of a longitudinal seminar to be taken up by other universities. Being transferable, it can be adapted to specific locations and situations. It can help to develop clinicians with excellent academic training at the service of society and empower them to make a positive contribution.

One of the participants puts the experienced impact of the LW in a nutshell:

*“The LW [learning workshop] has opened my eyes (…) that learning takes time but there is no need to neglect other aspects of life. I see that I can make changes, now I do no longer feel hung up in a downward cycle although the way to the top is still long.”* (Participant no. 15)

## Supplementary Information

**Additional file 1.** Evaluation Questionnaire LW.

**Additional file 2.** Consent of participation Evaluation LW.

## Data Availability

The datasets used and/or analyzed during the current study are available from the corresponding author on reasonable request. The students’ free-text answers are in German. There is no complete translation of all transcripts. Data was stored according to the agreements with participants and the ethical standards.
