# Supplementary material for: Fostering learning capacities for meaningful, healthy and efficient studying in undergraduate medical education: evaluation of a longitudinal learning workshop
Source: BMC Psychol. 2021 Sep 2;9:131. doi: 10.1186/s40359-021-00631-5 (PMC8414862; doi:10.1186/s40359-021-00631-5)
Supplement: Supplementary file 1 — Additional file 1. Evaluation Questionnaire LW. [file 40359_2021_631_MOESM1_ESM.pdf]

Learning Workshop (LW): Reflection and Evaluation

** mother: _ _ _ _ _ _ _ _ (DDMMYYYY)*

Personal reflection: My learning

1. Have you noticed any changes in your learning life due to your participation in the learning workshop?
    a. If yes, to what extent?

Learning Workshop (LW): Reflection and Evaluation

** mother: _ _ _ _ _ _ _ _ (DDMMYYYY)*

Personal evaluation: Satisfaction

2. How do you evaluate this change in general? (Satisfaction)

☐ „Yes, satisfied“: Why?

☐ „No, not satisfied“: Why not?

Learning Workshop (LW): Reflection and Evaluation

** mother: _ _ _ _ _ _ _ _ (DDMMYYYY)*

Learning workshop evaluation: Overall grade

3. The overall grade I give to the LW is:

(1) = Very Good
(2) = Good
(3) = Satisfactory
(4) = Sufficient
(5) = Poor
(6) = Deficient

Thank you for your participation!
