# Supplementary material for: Fostering learning capacities for meaningful, healthy and efficient studying in undergraduate medical education: evaluation of a longitudinal learning workshop
Source: BMC Psychol. 2021 Sep 2;9:131. doi: 10.1186/s40359-021-00631-5 (PMC8414862; doi:10.1186/s40359-021-00631-5)
Supplement: Supplementary file 2 — Additional file 2. Consent of participation Evaluation LW. [file 40359_2021_631_MOESM2_ESM.pdf]

Learning Workshop (LW): Reflection and Evaluation

Participants consent: Information

Purpose and Background

We are conducting an evaluation research study that is evaluating the Learning Workshop (LW). As part of the study, we are interested in students' perceptions of the LW in general and its impact on learning. We want to understand what did or did not work well to support contemporary learning. Your perspective will help us evaluate, suggest changes, and make recommendations to strengthen the efforts to improve the LW. The results from this study will be shared in a report or publication and used to optimize the LW and probably to inspire universities' didactical approach of learning.

Procedures

We are interested in learning about your experience in this area. You will be asked to participate by filling in the questionnaire at hand carefully. We like you to share your experiences and perceptions of the concept of the LW, the didactical approach and the potential impact on your learning life.

Risks

We do not anticipate any risks associated with participating in the study. If any question asked makes you uncomfortable, you are always free to decline to answer or to discontinue participation at any time.

Benefits

Participating in this study will give you an opportunity to add your experiences, ideas, opinions and recommendations around the LW.

Confidentiality

The researchers for this study will protect the confidentiality of whatever you share with them, and no identifying information will be released to anyone.

Compensation

There is no compensation for participating in this study.

Participation is Voluntary

You do not have to be in this study if you do not want to. You are free to decide how much time to invest in completing the questionnaire, how profound, detailed and precise your answers should be, and how much personal information you are willing to give. If you volunteer to be in this study, you may withdraw from it at any time without negative consequences for attendance at the LW nor potential proof of performance. By filling in the questionnaire and returning it to the researcher, you give permission to involve your evaluation in the research described at hand.

Contact Information

If you have any questions regarding your rights as a participant in this study, please contact: miriam.thye@uni-wh.de or diethard.tauschel@uni-wh.de

Witten/Herdecke University, Faculty of Health, Department of Medicine, Integrated Curriculum for Anthroposophic Medicine (ICURAM) and Department of Psychology and Psychotherapy

Thank you for your participation!

Diethard Tauschel & Miriam Thye
